# Ocean warming impairs the predator avoidance behaviour of elasmobranch embryos

**DOI:** 10.1093/conphys/coab045

**Published:** 2021-06-17

**Authors:** Daniel M Ripley, Sara De Giorgio, Kirstin Gaffney, Lowri Thomas, Holly A Shiels

**Affiliations:** Division of Cardiovascular Sciences, Faculty of Biology, Medicine and Health, Core Research Facility, The University of Manchester, 46 Grafton Street, Manchester M13 9NT, UK

**Keywords:** Climate change, conservation, freeze response, mechanical stimuli, metabolism, sharks

## Abstract

Embryogenesis is a vulnerable stage in elasmobranch development due in part to high predation mortality. Embryonic elasmobranchs respond to potential predators by displaying a freezing behaviour, characterized by the cessation of pharyngeal respiration followed immediately by coiling of the tail around the body. We hypothesized that the duration of this freeze response is limited by the embryo’s requirement for oxygen. Here, *Scyliorhinus canicula* embryos were incubated at either 15°C or 20°C during embryogenesis and tested for the duration of, and metabolic consequence of, the freeze response at their respective incubation temperature. Freeze response duration was negatively impacted by routine metabolic rate; embryos at 20°C had 7-fold shorter freeze duration than those at 15°C, potentially increasing their susceptibility to predation. These data demonstrate the capacity for climate change stressors to affect animal behaviour and suggest that this may occur by eliciting changes in the organism’s metabolism. We suggest altered predator avoidance behaviour is a new factor to consider when assessing the impact of climate change on the conservation and management of oviparous elasmobranch species.

## Teaser

Lay Summary

Shark embryos use a freezing behaviour to avoid being detected by predators. Here, we show that the duration an individual can perform the freezing behaviour is reduced by ocean warming, potentially increasing the vulnerability of embryonic sharks to predation.

## Introduction

Small-spotted catsharks (*Scyliorhinus canicula*) are oviparous elasmobranchs (Compagno, 1990), with embryos developing inside external, translucent, collagenous egg cases known colloquially as a mermaid’s purse. At ~15°C, *Scyliorhinus canicula* embryogenesis lasts ~21 weeks, with the embryo sealed inside its case for the first 10 weeks (Musa *et al*., 2018). Around week 10 (stages 4 and 31 on the Musa *et al*. 2018 and Ballard *et al*. 1993 scales, respectively) the hatching gland secretes digestive enzymes, opening slits in each of the egg cases’ 4 corners through which seawater enters (Ballard *et al*., 1993). Once the seawater slits are open, the embryo undulates rhythmically to actively exchange seawater to maintain the required oxygen saturation for continued development (Ballard *et al*., 1993, Leonard *et al*., 1999). This exchange of water, while providing the oxygen needed for growth, is also thought to release olfactory and mechanosensory cues from the egg case that predators may perceive (Kempster *et al*., 2013). Indeed, opening of the seawater slits and the onset of ventilatory undulation coincide with the development of both the electro- and mechano-sensory systems of the embryonic shark (Ballard *et al*., 1993), which can be used to detect predators (Sisneros *et al*., 1998). During exposure to a predator-mimicking bioelectric field, both thornback ray (*Raja clavata*) and brownbanded bamboo shark (*Chiloscyllium punctatum*) embryos respond by halting ventilatory behaviours and ceasing movement (Kempster *et al*., 2013, Ball *et al*., 2016), termed a freeze response. This freezing behaviour has also been observed in response to predator-like olfactory stimuli in embryonic Port Jackson sharks (*Heterodontus portjacksoni*) (Gervais *et al*., 2021). While the freezing behaviour has been demonstrated in several species of oviparous elasmobranch embryos in response to bioelectric-like (Kempster *et al*., 2013, Sisneros *et al*., 1998, Ball *et al*., 2016) and olfactory (Gervais *et al.*, 2021) stimuli, it is unknown whether this behaviour occurs in response to mechanical stimuli too. A substantial proportion of elasmobranch eggs are predated on by other sharks, often displaying signs of being crushed (Powter and Gladstone, 2008, Barrull and Mate, 2001). Therefore, it is likely that elasmobranch embryos respond to the mechanosensory, as well as the bioelectric, stimuli generated by predators. Regardless of the mechanism of induction, the duration that a freeze response can occur is finite, as eventually the individual will need to resume pharyngeal respiration and body undulations to ventilate the egg case (Leonard *et al*., 1999, Kempster *et al*., 2013, Di Santo *et al*., 2016). Thus, it is likely that an individual’s metabolic rate dictates the maximum duration, and consequently the effectiveness, of its freeze response.

Elasmobranchs are a major global conservation concern. Overfishing (particularly as by-catch), marine pollution and shark-finning have placed a quarter of all elasmobranch species under threat of extinction (Dulvy et al., 2014; Field et al., 2009; Fowler et al., 2005; Simpfendorfer and Dulvy, 2017). This is the lowest fraction of safe species across all extant vertebrates (Dulvy et al., 2014). These anthropogenic stressors are compounded by elasmobranch life history traits such as low fecundity rates, slow growth rates and delayed sexual maturity, which furthers their conservation concern. Early life stages of oviparous elasmobranchs have a particularly high mortality rate, rendering embryogenesis a critical intervention point for conservation and population management. Only 10% of Port Jackson shark (*Heterodontus portusjacksoni*) embryos survive until hatching, with 97.7% of the deaths being due to predation (Powter and Gladstone, 2008). Owing to this strong effect of predation on the survivorship of elasmobranch embryos, any environmental factors that alter the predator–prey interaction have the potential to greatly influence shark numbers (Myers *et al*., 2007) and thus warrant attention in relation to shark conservation.

In the past 100 years the earth’s climate has warmed by 0.6°C, with a further 1°C–3.7°C increase expected by 2100 (IPCC, 2014). As water temperatures rise, the metabolic rate of marine ectotherms increases. A study on juvenile Californian horn sharks (*Heterodontus francisci*) demonstrated a Q_10_ of 2.01 for their standard metabolic rate, implying a doubling of metabolic rate for a 10°C increase in water temperature (Luongo and Lowe, 2018). Similarly, the routine metabolic rate of elasmobranch embryos also increases with temperature, with brownbanded bamboo shark (*C. punctatum*) embryos demonstrating a Q_10_ between 3 and 5, depending on their developmental stage (Rosa *et al*., 2014). This positive relationship between environmental temperature and metabolic rate in marine ectotherms implies that future ocean warming will affect the efficacy of energetically costly, metabolism-limited behaviours, such as predator avoidance (McKenzie *et al*., 2016, Metcalfe *et al*., 2016, Cooke *et al*., 2003, Xia *et al*., 2021). However, this has not been explicitly tested in oviparous elasmobranch embryos. Here, we incubated *S. canicula* embryos at either 15°C or 20°C throughout embryogenesis and tested their behavioural and physiological response to a mechanical disturbance to assess the effect of ocean warming on an understudied, yet important, behaviour—the freeze response.

## Materials and Methods

### Embryo collection and incubation

All the experimental procedures and animal holding conditions were approved by the Animal Welfare and Ethics Review Board at The University of Manchester, UK. A total of 16 *S*. *canicula* embryos within the first 3 weeks of laying (stage 1, Musa *et al*., 2018; stages <16, Ballard *et al*., 1993) were shipped from the Ozeaneum, Stralsund, Germany, to The University of Manchester, UK. The breeding population of *S. canicula* at the Ozeaneum are maintained at 15°C. Upon arrival in Manchester, embryos were submerged in dechlorinated tap water for 2 min to remove any marine microorganisms and subsequently transferred to quarantine tanks at 15°C for 2 days. The health of each embryo was then checked following the protocol of Musa *et al*. (2020), following which healthy individuals were tagged and randomly assigned to either the 15°C or 20°C treatment group (*N* = 8 for 15°C; *N* = 8 for 20°C). Those assigned to 20°C were warmed from 15°C to 20°C at a rate of 2.5°C per day. The embryos were left in their tanks to develop until stage 6 (~15 weeks) of the Musa scale (stage 33 on the Ballard scale) before experimentation began (Musa *et al*., 2018, Ballard *et al*., 1993). This embryological stage was chosen as the mechanosensory system is developed, pharyngeal respiration is present and the embryos continually ventilate their egg case via body undulations (Musa *et al*., 2018, Ballard *et al*., 1993, Leonard *et al*., 1999). Each treatment group was split across two 55-l tanks to mitigate tank effects, which could not be assessed statistically owing to our modest N-number. The tanks were continuously aerated, and the temperature maintained by a heater connected to a temperature control unit (±0.3°C). A recirculating pump with inbuilt filters was used to maintain a clean environment and provide constant mixing to prevent thermal stratification. The water was tested three times per week for ammonia, nitrate and nitrite content, and water changes were performed accordingly to maintain them below detectable levels. Salinity was kept at 35 ppt. The mass of the embryo and its egg case at the beginning of the trials were not significantly different (7.34 g ± 0.56 g and 6.77 g ± 0.63 g; Mann–Whitney test, *P* = 0.1812) for the 15°C and 20°C groups, respectively.

### Ventilatory activity

Baseline ventilatory activity was measured in an aerated tank (width, 30 cm; length, 56 cm; depth, 30 cm) held at the embryo’s developmental temperature. Individual embryos were transferred in water, gently placed into the middle of the tank and then recorded for 10 min using a GoPro hero (GoPro, San Mateo, CA, USA). Ventilation frequency throughout the trial was measured in LoggerPro 3.14.1 (Vernier Software and Technology, Beaverton, USA). Preliminary analysis showed that ventilatory activity was suppressed for the first 2–3 min following handling, thus, the final ventilation frequency was calculated from minute 5 to minute 10 of each video.

### Freeze response duration, metabolic rates and cost of freezing

In the evening, embryos were taken from their holding tank in water, weighed and transferred to a circular test arena (radius, 15 cm; depth, 30 cm) containing water of the same temperature as their holding tank. Each embryo was handled underwater while simultaneously being flicked for 3 min to mimic the physical disturbances caused by predatory organisms investigating the egg case (Powter and Gladstone, 2008). Following this stressor, the embryo was quickly transferred in water to a custom-built, 150-ml plexiglass respirometry chamber to measure oxygen uptake as a proxy for metabolic rate (see below), and illuminated from underneath to allow visualization and recording (GoPro hero) of activity. The illumination was stopped 1-h post-stimulus and had no clear effect on the embryo’s rate of oxygen uptake. Freeze response duration was determined from the video footage as the time period between entering the chamber to the time that any buccal pumping or uncoiling of the tail occurred.

Embryos were left in the respirometry chamber overnight while oxygen uptake data were recorded. In total, data for each individual was recorded for 16 h. Each respirometry loop consisted of a 40-s flush, a 120-s wait period and a 740-s measurement phase. The flushing phase pushes oxygenated water into the otherwise sealed chamber (40 l hour^−1^, per chamber) preventing the system from becoming hypoxic and ensuring that the waste-products of metabolism do not accumulate (average O_2_ range (% air saturation) experienced in a respirometry loop: 15°C, 98.34%–94.25%; 20°C, 96.75%–90.32%). Following this, the flush pump turns off and a wait period begins in which the chamber is functionally sealed. During this time data are not recorded, allowing the newly flushed-in water to mix and reach equilibrium with the older water in the chamber. Finally, a measurement phase occurs in which the oxygen saturation of the chamber is recorded every second with a Firesting oxygen sensor (Pyroscience, Germany). The slope of the regression of oxygen content over time within each measurement phase is used as a proxy for the embryo’s metabolic rate. Only slopes with an R^2^ ≥ 0.9 were used in the final calculation of routine metabolic rate.

Routine metabolic rate was calculated as the mean oxygen uptake rate during the 16 h of measurement, excluding the first 60 min when the embryo was recovering from the freeze response. The oxygen uptake rate recorded during, and immediately following, the freeze response was calculated manually from the experiment’s beginning to the start of the first flush phase to account for the fact that an individual’s freeze response did not last one complete respirometry cycle. Immediately prior to, and following, the experiment the background oxygen uptake rate was measured. A mean background oxygen uptake rate was then calculated from these two measurements and subtracted from the total oxygen uptake rate of chamber. AquaResp 3.0 was used for the automation of the aquatic respirometry (University of Copenhagen; www.aquaresp.com)**.** Following the protocol each embryo was returned to its holding tank to continue embryogenesis.

Measurement of ventilatory activity, freeze response duration and oxygen uptake rates were performed blinded to reduce the potential influence of observer bias. However, as the freeze response was elicited by hand, we acknowledge that there is the potential for bias in this measurement.

### Data analysis

Routine metabolic rate, ventilation rate and freeze response duration were contrasted between the 15°C and 20°C embryos with Mann–Whitney tests to account for the relatively small N-numbers. The relationship between freeze response duration and routine metabolic rate was assessed using a linear model, with routine metabolic rate, environmental temperature and their interaction, as the predictors.

Oxygen uptake rate during, and in the 1 h following the freeze response, was converted to a percentage of the individual’s routine metabolic rate to normalize for both the effects of temperature and individual variation on routine metabolic rate. During the freeze response oxygen uptake rate is very low. Consequently, the subtraction of an average background oxygen uptake rate produced several negative values. As these negative values are not physiologically likely, and largely represent the effect of background correction, we constrained the values to ≥0 mgO_2_ hour^−1^. This only affected measurements during, but not after, the freeze response.

Breathing frequency in the 15 min following the stimulus was also converted to a percentage of each individual’s undisturbed breathing frequency for the same reasons. Owing to the size of the embryo relative the respirometry chamber, we found that we could not get robust oxygen uptake readings for measurement periods <30s, so this cut-off was set for measuring oxygen uptake during the freeze response. Due to the shorter freeze response duration of the sharks at 20°C, oxygen uptake measurements during the freeze response could only be calculated for 3/5 individuals at 20°C. All individuals were used in the analysis at all the other time points (*N* = 5 for the 15°C; *N* = 5 for 20°C groups). The post-stimulus metabolic and breathing rates, as a percentage of their undisturbed rates, were scaled to one and then arcsine transformed and contrasted between the 15°C and 20°C embryos using a mixed effects model with temperature, time point and their interaction as predictors, and individual ID as a random effect. Pairwise comparisons were performed using Šídák’s multiple comparisons test and false discovery rate corrected *P*-values were calculated. Mann–Whitney tests and mixed effects models were performed in GraphPad Prism 9.1.0, while linear models were computed in R 3.6.0. 

**Figure 1:**
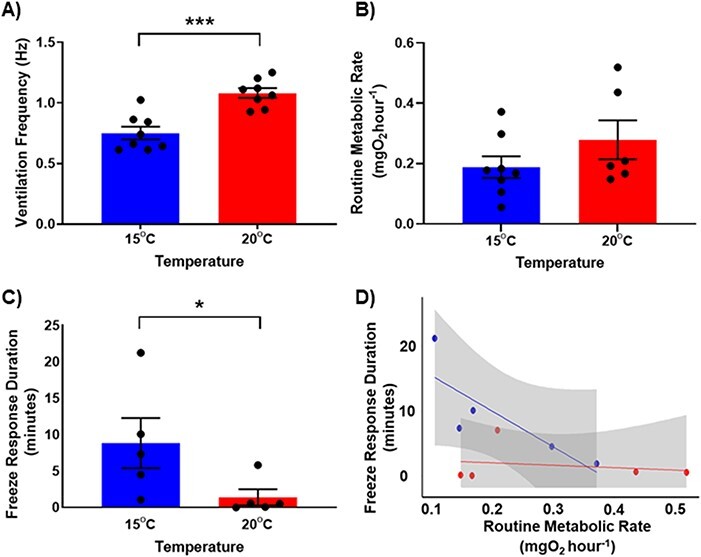
the **A**) ventilation frequency, **B**) routine metabolic rate, **C**) freeze response duration, and **D**) the relationship between routine metabolic rate and freeze response duration of *Scyliorhinus canicula* embryos tested at 15°C (blue) and 20°C (red); symbols denote individuals ±SEM A), *N* = 8 (15°C) & 8 (20°C); B), *N* = 8 (15°C) & 6 (20°C); C) *N* = 5 (15**°**C) & 5 (20°C); D), *N* = 5 (15**°**C) & 5 (20°C); the lines plotted were calculated from a linear model (*p* < 0.05); asterisks denote statistically significant differences between treatment groups (^*^p < 0.05, ^**^  *p* < 0.001, ^***^  *p* < 0.0001, Mann–Whitney test).

## Results

Ventilatory rate (Fig. 1A; Mann–Whitney test, *P* = 0.0006), but not routine metabolic rate (Fig. 1B; Mann Whitney test, *P* = 0.2284*)*, was greater in *S. canicula* embryos reared and tested at 20°C compared to those at 15°C. The duration of the freeze response was 7-fold shorter in embryos reared and tested at 20°C, compared to those from 15°C (Fig. 1C; Mann Whitney test, *P* = 0.0317). Freeze response duration was negatively affected by routine metabolic rate (Fig. 1D; linear model, *P* = 0.02667) and environmental temperature (Fig. 1D; linear model, *P* = 0.02474), but not their interaction (Fig. 1D; linear model, *P* = 0.06355).

**Figure 2:**
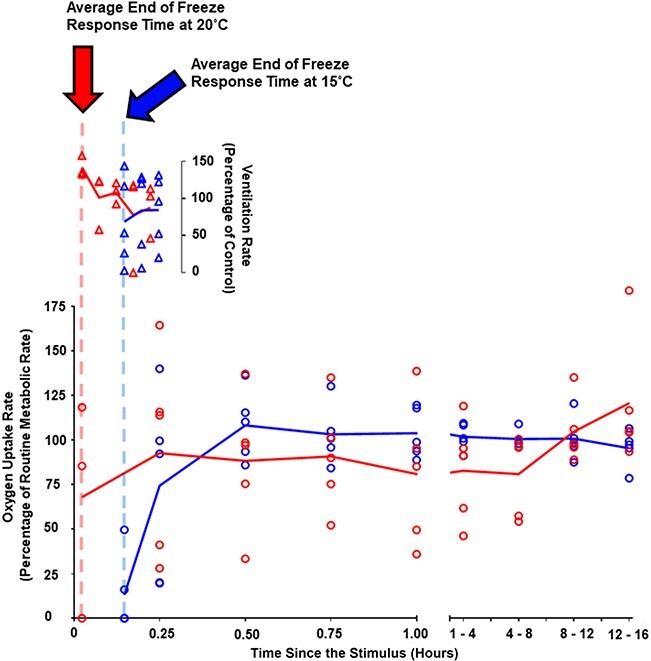
Lower panel is oxygen uptake as a percentage of routine metabolic rate (open circles) in the time post-stimulus, for sharks tested at 15°C (blue) and 20°C (red); oxygen uptake rates after the first one hour were binned into four groups to simplify data visualization; the dashed vertical lines represent the mean time at which the freeze response ended for the embryos tested at 15°C (dashed blue) and 20°C (dashed red) (raw data presented in Fig. 1C); oxygen uptake data at the first time point are plotted at each treatment group’s average freeze time; upper panel is breathing frequency as a percentage of control breathing rate (open triangles) following the resumption of breathing after a freeze; symbols denote individuals, and solid lines show the group means (*N* = 5 (15°C) & 3–5 (20°C)).

Oxygen uptake rate, as a percentage of routine metabolic rate, was significantly affected by time since the stimulus occurred (Fig. 2; mixed effects model, *P* = 0.0039), but not by temperature (Fig. 2; mixed effects model, *P* = 0.9777), or the interaction between temperature and time (Fig. 2; mixed effects model, *P* = 0.0956). Pairwise comparison testing revealed no significant difference between treatment groups in oxygen uptake rate at any given time point (Fig. 2; Šídák’s multiple comparisons test, FDR-corrected *P*-values > 0.05).

Post-stimulus breathing frequency, as a percentage of undisturbed breathing frequency, was not significantly affected by temperature (Fig. 2; mixed effects model, *P* = 0.2386) or time since the freeze response (Fig. 2; mixed effects model, *P* = 0.5450).

## Discussion

Previous studies have shown that elasmobranch embryos demonstrate freeze behaviour in response to stimuli mimicking the bioelectric fields and olfactory cues generated by fish (Kempster *et al*., 2013, Sisneros *et al*., 1998, Ball *et al*., 2016, Gervais *et al*., 2021). Here, we demonstrate that the same behaviour, characterized by a cessation of ventilatory movement (Kempster *et al*., 2013, Sisneros *et al*., 1998, Ball *et al*., 2016) followed immediately by rapid coiling of the tail around the body (Kempster *et al*., 2013), is induced by mechanical stimuli in *S. canicula* embryos. This is the first report of the freeze response behaviour in any elasmobranch as a response to ‘predatory’ mechanical stimuli. Importantly, we also demonstrate that the freeze response is 7-fold shorter under ocean warming conditions (+5°C), potentially increasing the vulnerability of embryonic elasmobranchs to predation. Finally, we show that the duration of an individual’s freeze response is influenced negatively by both its routine metabolic rate and environmental temperature.

The freeze response of embryonic elasmobranchs is thought to represent a mechanism to avoid detection by predators (Kempster *et al*., 2013). Undisturbed *S. canicula* embryos undulate rhythmically inside their egg case to draw in oxygenated water and expel the waste products of their metabolism (Leonard *et al*., 1999). However, while undulating the embryo is emitting visual, olfactory and physical signals revealing its whereabouts to potential predators. Upon detection of nearby organisms, *S. canicula* embryos cease pharyngeal respiration and undulating, curl their tail around their body and remain still in order to mask their presence from would-be predators. As oviparous elasmobranch embryos are sessile, this ability to ‘hide’ using the freeze response is likely crucial to their survival (Kempster *et al*., 2013).

The duration an individual can display the freezing behaviour may be limited by its capacity to maintain adequate oxygenation of its tissues while ceasing pharyngeal respiration and body undulations (Leonard *et al*., 1999, Kempster *et al*., 2013). A study on the common sole (*Solea solea*) demonstrated that in control conditions, but not under hypoxia, *S. solea* can greatly depress ventilatory activity when startled (Cannas *et al*., 2012), implying that this predator avoidance behaviour is limited by the requirement for adequate oxygen supply. Interestingly, we observed a negative relationship between the duration of an individual’s freeze response and their routine metabolic rate (Fig. 1D). However, despite *S. canicula* embryos not differing significantly in their routine metabolic rate at 15°C and 20°C, there was still an effect of environmental temperature on freeze response duration. Furthermore, ocean warming had no significant effect on the breathing frequency or oxygen uptake rates of embryonic sharks recovering from the freeze response (Fig. 2). Together, these data suggest that the mechanism(s) by which elevated temperature reduces freeze response duration in *S. canicula* may not be limited to aerobic metabolism alone.

Oceans are warming at an unprecedented rate. Representative concentration pathway (RCP) 8.5, a worst-case scenario model, delineates a world in which greenhouse gas emissions continue to rise throughout the century, causing ~3°C increase in mean ocean temperature (Cai *et al*., 2014). As mean ocean temperatures rise, both the frequency and duration of marine heatwaves increase, with an estimated 54% increase in annual marine heatwave days globally between 1925 and 2016 (Oliver *et al*., 2018). Together, these factors render it likely that some areas could experience 5°C above preindustrial levels before the end of the century. Thus, the negative effect of ocean warming on the freeze response behaviour may begin to affect the predator avoidance behaviour, and consequently the survival, of oviparous elasmobranch embryos. However, if rates of greenhouse gas emissions decline in accordance with RCP 2.6, mean ocean temperatures and the likelihood of marine heatwaves are estimated to plateau before the end of the century at an average temperature of <1°C above preindustrial levels (Cai *et al*., 2014).

Climate change and predation pressure are two major challenges facing oviparous elasmobranchs but are rarely considered together in conservation strategies. In some species, predation already accounts for up to 97.7% of embryonic mortality (Powter and Gladstone, 2008). Therefore, the ocean warming-induced reduction in duration of the anti-predator freeze response behaviour may exacerbate the already significant effect of predation on embryonic shark mortality. This potential reduction in shark recruitment could have consequences on ecosystem structuring as they often operate as apex or meso-predators, playing crucial roles as keystone species and top-down mediators of ecosystem function (Myers *et al*., 2007).

## Conclusion

Our study demonstrates that mechanical stimuli can elicit the freeze response behaviour in embryonic elasmobranchs. Furthermore, the data highlight ocean warming-induced changes in the freeze response behaviour of elasmobranch embryos, potentially increasing their susceptibility to predation. Future studies should expand the range of species and environmental stressors investigated, to develop a more thorough understanding of the mechanistic link between temperature, metabolism and the freeze response behaviour.
